# Targeting Human Epidermal Growth Factor Receptor 2 in Bladder Cancer: Evaluating Its Role as a More Robust Clinicopathological Biomarker Compared to Programmed Death Ligand 1 Expression

**DOI:** 10.5152/tud.2026.25061

**Published:** 2026-04-10

**Authors:** Ankur Mittal, Kunal Malhotra, Vikas Panwar, Sanjeev Kishore, Mohammed Taher, Avin Singhal

**Affiliations:** 1 Department of Urology, AIIMS Rishikesh, India; 2 Department of Pathology, AIIMS Rishikesh, India

**Keywords:** Biomarkers, HER2/neu, immunohistochemistry, immunotherapy, PD-L1, targeted therapy, urinary bladder neoplasms

## Abstract

**Objective:**

This retrospective cross-sectional analytical study evaluated the clinicopathological associations of programmed death ligand 1 (PD-L1) and human epidermal growth factor receptor 2 (HER2/neu) expression in 250 patients with urothelial carcinoma of the urinary bladder, with particular emphasis on their relationship to tumor stage and lymph node involvement.

**Methods:**

A retrospective observational study was conducted on 250 patients with urothelial carcinoma who underwent immunohistochemical evaluation of PD-L1, using a tumor proportion score (TPS) of ≥1%, and HER2/neu with 3+ considered positive. Formalin-fixed paraffin-embedded tissue from transurethral resection of bladder tumor and cystectomy specimens was analyzed by immunohistochemistry. The HER2/neu was scored using a standard 0-3+ system, and PD-L1 expression was assessed by TPS. Associations were tested using chi-square or Fisher’s exact tests. Multivariable logistic regression evaluated whether HER2/neu independently predicted nodal involvement after adjustment for age, sex, tumor stage, and morphology. Statistical significance was set at *P* < .05.

**Results:**

The HER2/neu 3+ positivity was present in 100/250 patients (40%) and was significantly associated with nodal involvement (*P* = .019). On multivariable logistic regression, HER2/neu is independently associated with nodal involvement, reflecting aggressive tumor biology (adjusted OR 2.41; 95% CI 1.33-4.36; *P* = .004). Among node-negative patients (N0, n = 200), 35.5% were HER2/neu positive, rising stepwise to 50.0% in N1, 54.5% in N2, and 71.4% in N3 disease, supporting a relationship between HER2/neu overexpression and nodal progression. In contrast, PD-L1 positivity (TPS ≥1%) was observed in 129/250 patients (51.6%) and was not significantly associated with age, sex, tumor stage, nodal status, grade, multiplicity, or morphology (all *P* > .05).

**Conclusion:**

The HER2/neu was an independent clinicopathological biomarker associated with nodal involvement and aggressive tumor biology in urothelial carcinoma. PD-L1 showed limited clinicopathological utility in this cohort, though it retains predictive value for immune checkpoint inhibitor therapy.

**Main Points**

- Human epidermal growth factor receptor 2 (HER2/neu) overexpression (3+) is significantly associated with advanced nodal involvement in urothelial carcinoma, supporting its association with tumor aggressiveness and nodal progression.
- Programmed death ligand 1 expression tumor proportion score (TPS ≥1%) showed no significant association with clinicopathological parameters in this cohort, indicating limited utility using TPS alone.
- The HER2/neu assessment may support risk stratification and patient selection for clinical trials of HER2-targeted strategies in urothelial carcinoma.

## Introduction

Urothelial carcinoma of the urinary bladder is among the most common malignancies of the urinary tract and is associated with substantial morbidity and mortality worldwide.^1^ Despite advances in diagnosis and treatment, clinical outcomes remain heterogeneous, largely reflecting the biological diversity of the disease.^2^ Molecular characterization has identified biomarkers such as programmed death ligand 1 (PD-L1) and human epidermal growth factor receptor 2 (HER2/neu), which may influence tumor behavior, therapeutic response, and patient management.^3^

The HER2/neu and PD-L1 have emerged as important molecular targets in bladder cancer. HER2/neu, well established in breast and gastric cancers with successful targeted therapies such as trastuzumab,^4^ is expressed in a subset of urothelial carcinomas and has been associated with aggressive tumor behavior, higher stage, and poor pathological features, although results across studies remain inconsistent.^5^^,^^6^ Immune checkpoint inhibitors targeting the PD-1/PD-L1 axis, including pembrolizumab and atezolizumab, have improved outcomes in selected patients with advanced urothelial carcinoma.^7^^,^^8^ However, heterogeneity in PD-L1 expression and response to immunotherapy limits its utility as a standalone biomarker.^9^ Emerging data suggest potential biological interactions between HER2 and PD-L1 expression, which may further influence tumor behavior and therapeutic response.^10^

Recent systematic reviews indicate that HER2 positivity is more frequently associated with higher tumor grade, advanced stage, and nodal metastasis in urothelial carcinoma and upper tract disease, supporting its role as a marker of aggressive pathology.^11^^,^^12^ In contrast, the clinicopathological significance of PD-L1 expression remains controversial; a recent meta-analysis demonstrated that PD-L1 positivity was associated with improved response and survival outcomes in patients receiving immune checkpoint inhibitors, but its predictive and clinicopathological value remains inconsistent.^13^ Direct comparisons of HER2/neu and PD-L1 expression with respect to nodal involvement are limited, particularly in studies without survival follow-up. Accordingly, this retrospective cross-sectional analytical study aimed to evaluate and compare the clinicopathological associations of PD-L1 and HER2/neu expression in a cohort of 250 patients with urothelial carcinoma, with specific focus on tumor stage and lymph-node involvement (Table 1).

This study aimed to compare the clinicopathological significance of PD-L1 and HER2/neu expression and to determine whether HER2/neu independently predicts lymph node involvement in a cohort of 250 patients with urothelial carcinoma (Table 2).

## Material and Methods

### Study Design and Patient Selection

This was a retrospective cross-sectional analytical study evaluating biomarker expression and clinicopathological parameters at the time of diagnosis. Ethical approval was obtained from the All India Institute of Medical Sciences (AIIMS), Rishikesh, India Institutional Ethics Committee prior to retrieval of anonymized clinical and pathological data via letter no -AIIMS/IEC/25/026 dated March 16, 2025. Patient consent was waived due to the retrospective nature of the study and use of anonymized data, as approved by the Institutional Ethics Committee.

Patients diagnosed between January 2019 and December 2024 were identified from institutional histopathology archives and corresponding medical records. All cases had undergone routine diagnostic evaluation and treatment during this period. No prospective enrollment or additional follow-up was undertaken as part of this study.

Inclusion criteria: Histologically confirmed urothelial carcinoma with availability of formalin-fixed paraffin-embedded (FFPE) tissue blocks suitable for immunohistochemistry.

Exclusion criteria: Incomplete clinical records or inadequate tissue for immunohistochemical assessment.

#### Tissue Samples and Histopathology Review:

The FFPE tissue blocks from transurethral resection of bladder tumor or cystectomy specimens were retrieved. Hematoxylin and eosin–stained slides were reviewed by a pathologist to confirm diagnosis and to select representative tumor areas. Tissue microarray blocks were constructed using 2 1.5-mm cores from representative tumor regions of each case.

#### Immunohistochemical Analysis:

Immunohistochemistry was performed on 3-4 µm sections. Following deparaffinization and rehydration, antigen retrieval was performed using heat-induced epitope retrieval: citrate buffer (pH 6.0) for HER2/neu and Tris-EDTA buffer (pH 9.0) for PD-L1. Endogenous peroxidase was blocked with hydrogen peroxide. Sections were incubated with primary antibodies for HER2/neu and PD-L1, followed by secondary antibody and streptavidin-HRP. 3,3 - diaminobenzidine (DAB) was used for visualization and slides were counterstained with hematoxylin.

Scoring:

- HER2/neu: scored 0–3+; 3+ considered positive.
- PD-L1: Tumor proportion score (TPS) is defined as the percentage of viable tumor cells with membranous staining; TPS ≥1% considered positive.

### Statistical Analysis

Data were collected and analyzed using SPSS, version 22.0 (IBM SPSS Corp.; Armonk, NY, USA). Categorical variables were compared using chi-square or Fisher’s exact tests. Multivariable logistic regression assessed whether HER2/neu positivity independently predicted nodal involvement after adjustment for age, sex, tumor stage, and morphology. Adjusted odds ratios (aORs) with 95% CIs were reported. No formal correction for multiple comparisons was applied; marginal associations were interpreted cautiously. Statistical significance was defined as *P* < .05.

## Results

### Cohort Characteristics

The cohort included 176 males (70.4%) and 74 females (29.6%), with a mean age of 58.3 ± 11.2 years (range 28-82). Muscle-invasive disease (≥T2) was present in 132 patients (52.8%) and non-muscle-invasive disease in 118 (47.2%). High-grade histology was observed in 186 patients (74.4%) and low-grade disease in 64 (25.6%). Nodal involvement was present in 84 patients (33.6%), comprising N1 (n = 38), N2 (n = 26), and N3 (n = 20).

### Programmed Death-Ligand 1 Expression and Clinicopathological Associations

Programmed death-ligand 1 positivity (TPS ≥1%) was observed in 129/250 patients (51.6%); 121 (48.4%) were PD-L1 negative.

The PD-L1 expression showed no statistically significant association with age, sex, tumor stage, nodal status, tumor grade, multiplicity, or morphology (all *P* > .05). Similar PD-L1 positivity rates were observed across muscle-invasive and non-muscle-invasive tumors and across grade subgroups, indicating limited clinicopathological relevance in this cohort.

### Human Epidermal Growth Factor Receptor 2 Expression and Nodal Progression

The HER2/neu 3+ positivity was present in 100/250 patients (40%). The HER2/neu positivity was significantly associated with nodal involvement (*P* = .019) and demonstrated a stepwise increase with advancing nodal stage.

For nodal subgroup analysis, complete nodal staging and immunohistochemical data were available for 200 node-negative (N0) patients and 50 node-positive patients (N1-N3). Among N0 patients (n = 200), 35.5% were HER2/neu positive. The HER2/neu positivity increased progressively with nodal disease: 50.0% in N1 (n = 14), 54.5% in N2 (n = 22), and 71.4% in N3 (n = 14), supporting a strong association between HER2/neu overexpression and nodal progression.

The HER2/neu positivity was more frequent in high-grade tumors than low-grade tumors (44.1% vs. 28.1%), though this did not reach statistical significance. No significant association was observed between HER2/neu and tumor multiplicity or tumor morphology.

### Multivariable Logistic Regression

**Figure 1.**
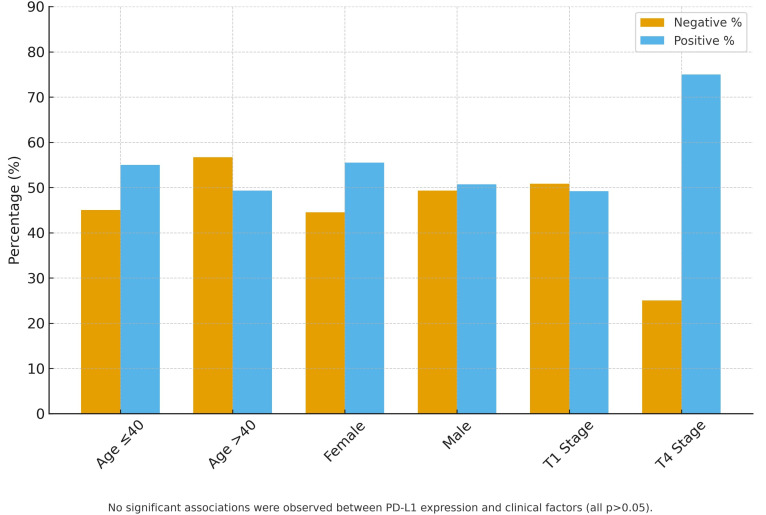
Association between PD-L1 expression and clinicopathological factors in urothelial carcinoma (n = 250).

**Figure 2.**
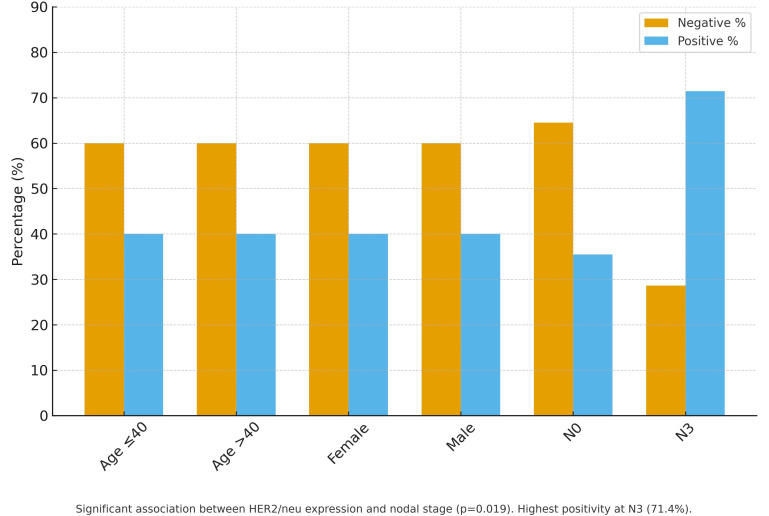
Association between HER2/neu expression and clinicopathological factors in urothelial carcinoma (n = 250).

On multivariable logistic regression, HER2/neu 3+ expression remained an independent predictor of nodal involvement after adjustment for age, sex, tumor stage, and morphology (aOR 2.41; 95% CI 1.33-4.36; *P* = .004). Further subgroup analysis stratified by age (<40 years vs. ≥40 years) was performed to evaluate whether the clinicopathological relevance of HER2/neu expression differed between early-onset and conventional-onset urothelial carcinoma. The association between HER2/neu expression and aggressive pathological features according to age group is shown in Table 3 [Table t2-urp-52-1-25061]and Figures 1 and 2.

The model showed good calibration (Hosmer–Lemeshow, *P* = .61) and acceptable discrimination (AUC, 0.72). The PD-L1 was not included in the multivariable model because it did not show meaningful association with nodal involvement or other aggressive pathological features on univariate testing.

### Age-Stratified Subgroup Analysis

In patients aged <40 years, HER2/neu positivity was not significantly associated with nodal involvement (*P* = 1.000) or muscle-invasive disease (*P* = .287), likely reflecting limited sample size and low event rates. In patients aged ≥40 years, HER2/neu positivity showed a significant association with nodal involvement (*P* = .015), while no significant association with muscle invasion was observed (*P* = .583).

Overall, HER2/neu overexpression showed a clear relationship with nodal progression in the overall cohort and in patients aged ≥40 years, whereas PD-L1 expression was not associated with clinicopathological parameters analyzed.

## Discussion

In this retrospective study of 250 patients with urothelial carcinoma, HER2/neu 3+ expression was significantly associated with nodal involvement and remained an independent predictor of nodal disease after adjustment for relevant covariates. The proportion of HER2/neu positivity increased stepwise with nodal stage, supporting an association between HER2/neu overexpression and nodal progression. These findings align with literature indicating that HER2/neu overexpression may reflect aggressive tumor biology in urothelial carcinoma.^5^^,^^13^^,^^14^

By contrast, PD-L1 (TPS ≥1%) was not associated with age, sex, stage, nodal status, grade, multiplicity, or morphology in the cohort, indicating limited clinicopathological utility using TPS alone. This is consistent with prior observations that immune checkpoint pathway marker expression in muscle-invasive urothelial carcinoma can be independent of demographic factors such as age and sex.^12^ Recent cohorts have also reported variable associations between PD-L1 expression and clinicopathological variables, reflecting differences in tumor microenvironment, assay platforms, and scoring systems.^11^^,^^15^

The findings support a model in which HER2/neu more directly reflects intrinsic tumor aggressiveness and metastatic potential, whereas PD-L1 expression may be influenced by microenvironmental immune context and dynamic immune–tumor interactions.^7^^,^^8^ Reports describing associations between HER2/neu and PD-L1 expression suggest potential biological interplay, though the direction and clinical implications remain incompletely defined.^10^^,^^11^ Comprehensive biomarker frameworks integrating tumor-intrinsic and immune markers are increasingly emphasized in urothelial carcinoma.^7^

Although the study demonstrates a robust relationship between HER2/neu 3+ status and nodal involvement, survival outcomes were not evaluated. Therefore, direct confirmation of HER2/neu as a survival prognostic biomarker in this cohort cannot be established. Prior survival analyses have produced mixed signals; notably, 1 study reported an association between HER2 overexpression and overall survival in muscle-invasive bladder cancer undergoing cystectomy, underscoring clinical relevance while also highlighting heterogeneity across cohorts.^15^ Prospective validation in independent cohorts with long-term outcomes is needed.

### Limitations

This study is limited by its retrospective single-center design, which may affect generalizability. PD-L1 scoring was restricted to TPS ≥1%; alternative scoring systems such as combined positive score or immune-cell scoring were not applied. Treatment-specific predictive analyses for immunotherapy or HER2-targeted therapy could not be performed because patients did not receive these agents as part of routine care during the study period. Finally, subgroup analyses (e.g., <40 years) were underpowered due to limited sample size and event rates.

In this cohort, PD-L1 expression (TPS ≥1%) showed no significant association with clinicopathological parameters, suggesting limited value when used as a standalone marker. In contrast, HER2/neu 3+ expression was significantly associated with nodal involvement and independently predicted nodal disease after adjustment for covariates, supporting its relevance as a marker of aggressive tumor biology. While PD-L1 remains important as a predictive biomarker for immune checkpoint inhibitor therapy, HER2/neu appears more clinically informative for identifying tumors with higher metastatic potential. Prospective studies incorporating survival outcomes and treatment response are required to validate these findings and define the role of HER2/neu testing in routine clinical practice.

## Figures and Tables

**Table 1.** Association of PD-L1 Expression with Clinicopathological Parameters

**Parameter**	**Negative (n = 121) (%)**	**Positive (n = 129) (%)**	***P***
Age group			
Up to 40 years (n = 100)	45 (45)	55 (55)	.38
>40 years (n = 150)	76 (56.7)	74 (49.3)	
Gender			
Female (n = 45)	20 (44.5)	25 (55.5)	.558
Male (n = 205)	101 (49.3)	104 (50.7)	
Papillary/solid			
Architecture			
Papillary (n = 26)	12 (46.2)	14 (53.8)	.808
Solid (n = 224)	109 (48.6)	115 (51.4)	
Multiple/single lesion			
Multiple (n = 84)	41 (48.8)	43 (51.2)	.926
Single (n = 166)	80 (48.2)	86 (51.8)	
Tumor stage			
T1 (n = 65)	33 (50.8)	32 (49.2)	.743
T2 (n = 169)	82 (45.2)	87 (51.8)	
T3 (n = 12)	5 (41.7)	7 (58.3)	
T4 (n = 4)	1 (25.0)	3 (75.0)	
Nodal stage			
N0 (n = 200)	100 (50.0)	100 (50.0)	0.716
N1 (n = 14)	5 (35.7)	9 (64.3)	
N2 (n = 22)	10 (45.5)	12 (54.5)	
N3 (n = 14)	6 (42.9)	8 (57.1)	

*P *values calculated using chi-square test or Fisher’s exact test where appropriate.

**Table 2. t2-urp-52-1-25061:** Association of HER2/neu Expression with Clinicopathological Parameters

**Parameter**	**Negative (n = 150) (%)**	**Positive (n = 100) (%)**	***P***
Age group			
Up to 40 years (n = 100)	61 (61.0)	39 (39.0)	0.792
>40 years (n = 150)	89 (59.3)	61 (40.7)	
Gender			
Female (n = 45)	27 (60.0)	18 (40.0)	1.000
Male (n = 205)	123 (60.0)	82 (40.0)	
Papillary/solid architecture			
Papillary (n = 26)	15 (57.7)	11 (42.3)	1.000
Solid (n = 224)	135 (60.3)	89 (39.7)	
Multiple/Single lesion			
Multiple (n = 84)	51 (60.7)	33 (39.3)	.870
Single (n = 166)	99 (59.6)	67 (40.4)	
Tumor stage			
T1 (n = 65)	32 (49.3)	33 (50.7)	.088
T2 (n = 169)	106 (62.7)	63 (37.3)	
T3 (n = 12)	10 (83.3)	2 (16.7)	
T4 (n = 4)	2 (50.0)	2 (50.0)	
Nodal stage			
N0 (n = 200)	129 (64.5)	71 (35.5)	.019
N1 (n = 14)	7 (50.0)	7 (50.0)	
N2 (n = 22)	10 (45.5)	12 (54.5)	
N3 (n = 14)	4 (28.6)	10 (71.4)	

*P* values calculated using chi-square test or Fisher’s exact test, as appropriate.

**Table 3.** Association of HER2/neu Expression with Aggressive Features by Age Group

**Feature**	**Age Group**	**HER2+ (%)**	**HER2− (%)**	***P***
Nodal involvement	<40 years	2/7 (28.6)	4/21 (19.0)	1.000
Nodal involvement	≥40 years	18/62 (29.0)	17/128 (13.3)	.015
Muscle invasion (≥T2)	<40 years	4/7 (57.1)	18/21 (85.7)	.287
Muscle invasion (≥T2)	≥40 years	44 /62 (71.0)	9 /128 (72.7)	.583

*P* values calculated using Fisher’s exact test due to small subgroup sample sizes.

## Data Availability

The data that support the findings of this study are available on request from the corresponding author.
